# Development of a health related quality of life questionnaire for adult patients with Primary Ciliary Dyskinesia

**DOI:** 10.1186/2046-2530-1-S1-P6

**Published:** 2012-11-16

**Authors:** JS Lucas, NJ Botting, A DunnGalvin, F Copeland

**Affiliations:** 1 University of Southampton, UK; 2 University of Cork, Ireland; 3 PCD Family Support Group, UK

## Background

Primary Ciliary Dyskinesia (PCD) is a rare inherited disease which causes chronic lung disease, sinusitis, rhinitis, glue ear and often sub-fertility. We are developing a health-related quality of life (QoL) questionnaire for adults with PCD, to monitor long term outcomes and for use in clinical trials.

## Methods

Seventy-eight items important for QoL in PCD were generated by patients, health-specialists and a literature review. To reduce the list to the most relevant items, it was sent to adult members of the PCD Support Group, European PCD Taskforce and Southampton patients. 49 questionnaires were returned, with each item ranked on a 5-point Likert scale (1=not at all relevant, 5=very relevant). Mean values for each item were calculated.

## Results

The emotional impact of the disease ranked highly. For example, ‘Performing physiotherapy in front of others causes embarrassment’ had the highest mean score (4.26), whilst the ‘therapy is not enjoyable’ was less of an issue (3.50).Concern about the future and about always requiring treatment for PCD, lack of understanding about PCD by others and the symptom of coughing were highly relevant (all >4.00). Several items were deemed to be irrelevant, such as those concerned with seeing doctors on a regular basis and impact on social life.

## Conclusions

Patients reported greater impact from emotional consequences and embarrassment about PCD and its treatment, than from the physical or social consequences. The prototype questionnaire with the most relevant items is nearing completion. Full cross-sectional and longitudinal validation will be conducted in UK, Ireland and USA.

